# Utilization of Tamarind, Pineapple, and Chickpea Pulp as Sources of Bioactive Compounds in a Functional Confectionery: Nutritional Composition, Antioxidant Potential, and In Vitro Digestibility

**DOI:** 10.3390/foods14233987

**Published:** 2025-11-21

**Authors:** Alma Cristina Gaytán-Lara, Héctor Emmanuel Cortés-Ferré, Aurea Karina Ramírez-Jiménez, Elisa Dufoo-Hurtado, Mar Villamiel, Marcela Gaytán-Martínez

**Affiliations:** 1 Posgrado de Alimentos, Facultad de Química, Universidad Autónoma de Querétaro, Santiago de Querétaro 76177, Querétaro, Mexico; alma.ga.la98@gmail.com (A.C.G.-L.); hcortesf@tec.mx (H.E.C.-F.); 2 Departamento de Ingeniería Química Industrial y de Alimentos, Universidad Iberoamericana Ciudad de México, Prolongación Paseo de la Reforma 880, Ciudad de México 01219, Mexico; 3 Campus Querétaro, Tecnológico de Monterrey, Santiago de Querétaro 76130, Querétaro, Mexico; aramirezj@tec.mx (A.K.R.-J.);; 4 Instituto de Investigación en Ciencias de la Alimentación, CIAL (CSIC-UAM), Campus de la Universidad Autónoma de Madrid, 28049 Madrid, Spain

**Keywords:** tamarind, confectionery, functional

## Abstract

Confectionery products are typically rich in refined sugars and poor in nutrients. This study explored the incorporation of tamarind (*Tamarindus indica*), pineapple (*Ananas comosus*), and chickpea (*Cicer arietinum*) pulps as sources of bioactive compounds in functional soft candies. Two optimized formulations (33T/33G/33P and 66T/16G/16P) were developed based on consumer acceptability and texture profile rather than taste imitation, both formulations achieved good sensory acceptance (scores above 7 on a 9-point scale, *p* < 0.05), confirming their suitability for functional product development. Compared to conventional candies, the functional confections exhibited up to threefold higher protein and fiber contents (13.6% and 32%, respectively) and markedly enhanced antioxidant potential. The formulation enriched with tamarind (66T/16G/16P) showed the highest total phenolics (22.39 mg GAE/g) and antioxidant capacity (27.36 µM Trolox eq/g). Among the eight phenolic compounds identified by HPLC, catechin (2.72 ± 0.72 mg/g) and epicatechin (53.22 ± 2.68 mg/g) were the main preserved components. In vitro digestion indicated moderate protein bio-accessibility, likely influenced by protein–phenolic interactions. Overall, the results highlight the potential of integrating fruit and legume pulps into confectionery matrices to create nutrient-enriched, plant-based alternatives to traditional sweets.

## 1. Introduction

Typically, consumer demand for healthier and more sustainable food options has driven the food industry to reformulate traditional confectionery products, which are typically high in refined sugars and low in nutrients [1]. These products, while widely consumed, contribute to nutritional imbalances and chronic health conditions. In this context, functional confectionery—sweets designed to provide physiological benefits beyond basic nutrition—has become a rapidly expanding research area.

Previous studies have demonstrated the potential of functional chocolates, gums, and chewy candies as carriers of bioactive compounds or natural ingredients [2,3]. For example, functional chocolates enriched with antioxidants have been shown to protect against oxidative damage and support liver function [2], while chewing gums have been explored as delivery systems for bioactive that reduce stress or enhance cognitive performance [4]. Similarly, fruit-based chewy candies formulated with guava, mango, or molasses have proven to be attractive alternatives to conventional sweets by eliminating artificial colorants and lowering refined sugar content [3,5].

Despite these advances, most studies have focused on single fruit matrices, overlooking the synergistic potential of combining different plant-based ingredients with complementary nutritional profiles. Tamarind (*Tamarindus indica*), pineapple (*Ananas comosus*), and chickpea (*Cicer arietinum*) each represent valuable sources of phenolic compounds, dietary fiber, and proteins, respectively, but their joint use in confectionery formulations has not yet been explored.

Tamarind pulp is a rich source of polyphenols (flavonoids, tannins, and phenolic acids) with reported antioxidant, antihyperglycemic, and hepatoprotective effects [6,7]. Pineapple pulp contributes vitamin C and bromelain; a proteolytic enzyme associated with anti-inflammatory and digestive properties [8]. Chickpea flour, in turn, provides plant-based proteins with moderate digestibility (34–76%) and high fiber content (18–22%), along with isoflavones and phenolic acids that support anti-inflammatory and antidiabetic effects [9].

The combination of these three ingredients was hypothesized to exert synergistic effects due to their complementary bioactive profiles. Tamarind and pineapple pulps are rich in polyphenols and enzymatic compounds such as bromelain, which contribute to antioxidant and anti-inflammatory activities. Chickpea flour, in contrast, provides plant-based proteins and soluble dietary fiber capable of forming non-covalent complexes with phenolics. These interactions may enhance the stability of phenolic compounds during processing and digestion, improving their bio-accessibility and antioxidant potential. Therefore, the integration of tamarind, pineapple, and chickpea offers a balanced formulation that unites phenolic-rich fruits with a protein- and fiber-based matrix, supporting the design of multifunctional confectionery products with controlled release of bioactives and improved nutritional quality.

Therefore, the present study aims to (i) formulate functional soft candies incorporating tamarind, pineapple, and chickpea pulps; (ii) evaluate their proximate composition, phytochemical profile, and antioxidant capacity; and (iii) assess their in vitro protein digestibility to understand nutrient bio-accessibility. By addressing the gap in the literature regarding multi-ingredient, plant-based functional candies, this work contributes to the design of confectionery products with improved nutritional and bioactive properties, supporting innovation in healthier sweet formulations.

## 2. Materials and Methods

### 2.1. Raw Materials

Tamarind was purchased at the Querétaro state supply center. The peel was manually removed, and the fruit was soaked for 31 min at 38 °C at a 2.5:1 ratio (water: fruit) [10]. After soaking, the fruit was pulped using a Polinox pulper. The tamarind purchased contained 5.09 ± 0.02% reducing sugars, 49.33 ± 0.58° Brix, and 11.10% tartaric acid.

The pineapple was donated by “Piñas Del Pacífico S.P.R. de R.L.”, washed, disinfected with chlorinated water (sodium hypochlorite at 50–100 ppm), and then crushed [11]. The fruit used contained 6.51 ± 0.34% reducing sugars, 11.97 ± 0.12° Brix, and 1.59 ± 0.003% citric acid.

For chickpeas, extruded chickpea flour (180 °C with screw speed of 15 rpm), previously prepared by our research team [12], was used. This study demonstrated that under these extrusion conditions, the flour exhibited improved techno-functional properties including foam stability, gelling capacity, and viscosity. Furthermore, the treatment reduced the antinutritional compounds in the chickpea without affecting its in vitro digestibility. The flour used in this research contained 19.80 ± 0.44% protein, 23.20 ± 0.15% total fiber (dry basis), and a moisture content of 6.44 ± 0.55%.

After processing, the tamarind and pineapple pulp were frozen at −18 °C until the candy was made.

### 2.2. Materials

Kjeldahl catalyst tablets (BUCHI Labortechnik AG, Flawil, Switzerland) were used. Folin–Ciocalteu reagent was purchased from Sigma-Aldrich (St. Louis, MO, USA). The HPLC standards used were purchased from Sigma-Aldrich (St. Louis, MO, USA): gallic acid, catechin, vanillic acid, epicatechin, ferulic acid, hesperidin, vanillin, rutin, p-coumaric acid, quercetin, naringenin, and caffeic acid. For the in vitro digestibility study, α-amylase from human saliva (500 UI/mg, Sigma-Aldrich, St. Louis, MO, USA), pepsin from porcine gastric mucosa (400 UI/mg, Sigma-Aldrich, St. Louis, MO, USA), lipase from porcine pancreas (100–500 UI/mg, Sigma-Aldrich, St. Louis, MO, USA), pancreatin from porcine pancreas (8 × USP, Sigma-Aldrich, St. Louis, MO, USA), and bile extract porcine (Sigma-Aldrich, St. Louis, MO, USA) were used. The solvents used in HPLC (methanol, water, acetonitrile and formic acid) were HPLC grade (Sigma-Aldrich, St. Louis, MO, USA).

### 2.3. Manufacturing Process

An experimental mixture design was used to prepare the functional candy (Table 1). Chickpea flour was first dispersed in 70% water and heated to 65 °C for 2 min with constant manual stirring. Tamarind and pineapple pulps were then incorporated, followed by 0.3% pre-hydrated xanthan gum and 3% commercial chili powder (Hill Country Fare, San Antonio, TX, USA). The mixture was reheated to 65 °C for another 2 min until homogeneous. After cooking, the samples were cooled to 25 °C and stored at 4 °C.

All formulations and commercial candy were subjected to instrumental texture profile analysis (TPA: hardness, adhesiveness, cohesiveness, springiness) and oscillatory rheology (storage modulus G′ and loss modulus G″) to identify formulations with mechanical behavior like the commercial reference. Samples 7 (33% tamarind, 33% pineapple, 33% chickpea) and 8 (66% tamarind, 16% pineapple, 16% chickpea) exhibited the closest rheological and textural profiles and were therefore selected for further analyses.

The complete dataset describing the rheological and sensory optimization process is part of a complementary study currently under peer review by our research group and supports the selection of samples 7 and 8 as representative formulations. The present work focuses on the nutritional composition, bioactive properties, and in vitro digestibility of the two optimized formulations.

### 2.4. Microbiological Analysis

According to a previous study [13], it is essential to determine *Salmonella* spp. in this type of product. Coliform bacteria were also determined using the Most Probable Number (MPN) technique [14] and aerial mesophylls by plate method [15]. This study was conducted solely to ensure product safety for subsequent sensory evaluation. Once the microbiological criteria established by Mexican regulations [16] were met, the sensory analysis proceeded.

### 2.5. Sensory Analysis

Before the proximate and phytochemical characterization of the samples, a sensory analysis was performed. This analysis was conducted to assess consumer acceptance, which is an essential parameter for developing a new product.

The sensory analysis was conducted by recruiting 55 untrained panelists aged 18 to 40 years of age through an open invitation. They were required to meet the following criteria for participation: not to have allergies or intolerances to tamarind, pineapple, chickpeas, or chili, and to be a regular consumer of tamarind sweets. Participation was free of charge. Before the analysis, panelists were given an informed consent form.

The samples were presented at room temperature (25 °C) in a random and balanced manner, without artificial lighting, and were coded with random 3-digit numbers. They were prepared within the same time frame and in the same type of container.

The test consisted of two parts. First, a quantitative acceptance test was conducted using a 9-point hedonic scale, where 1 indicated “I dislike it very much” and 9 indicated “I like it very much”. In this test, the panelists received two 10 g samples, rinsing them with water and a neutral cookie between each evaluation. After the first part, they conducted a preference test alongside a control sample, ranking the samples from 1 (most preferred) to 3 (least preferred). The results were analyzed using the nonparametric Kruskal–Wallis test.

Researchers performed both tests in the sensory analysis laboratory at Universidad Autónoma de Querétaro.

### 2.6. Bromatological Characterization of Confectionery Products

The samples with the highest sensorial acceptance by the panelists and a commercial sample (as control) were subjected to a bromatological characterization following the methods described by the AOAC (2005) [17]: moisture (method 325.23), proteins (method 920.15), ashes (method 942.05), lipids (method 920.39), dietary fiber (method 991.43).

### 2.7. Phytochemical Characterization

The same samples were characterized by bromatological and phytochemical methods. The extracts were prepared according to Dávila-Hernández et al. (2020) [18] with slight modifications. A 0.5 g dry sample was weighed and dissolved in 10 mL of 80% HPLC-grade methanol, then left to stand for 12 hr. Subsequently, it was sonicated for 54 min at 44 °C, followed by centrifugation at 5000 rpm for 10 min, after which the supernatant was recovered.

### 2.8. Phenolic Compounds

Total Phenolic Compound quantification was performed using the Folin–Ciocalteu method [19], with some modifications. In plant extracts, compounds such as reducing sugars and vitamin C can interfere with the determination of total phenolic compounds [20]. For this reason, a pretreatment with 1.5 mol/dm^3^ hydrogen peroxide was carried out [21]. After the pretreatment, 50 µL of the extracts were transferred to a 2 mL Eppendorf tube. 250 µL of distilled water and 125 µL of Folin reagent (1 eq/L) were added, and the mixture was shaken. Finally, 625 µL of 7% Na_2_CO_3_ were added. It was incubated for 2 h at 27 °C and in the dark. After the specified time had elapsed, 250 µL were taken and placed in a 96-well microplate to measure the absorbance at 760 nm using a previously calibrated microplate spectrophotometer (Multiskan Skyhigh, Thermo Fisher Scientific, Waltham, MA, USA). The calibration curve was obtained by linear regression from seven levels of gallic acid concentration measured in triplicate, with a correlation coefficient (R^2^) of 0.9907. The results were expressed as mg of gallic acid equivalents per gram of dry sample.

### 2.9. Condensed Tannins

It was determined by mixing 50 μL of extract in 200 μL of 1:1 8% HCl:1% vanillin solution [22]. Absorbance was read at 492 nm using a previously calibrated microplate spectrophotometer (Multiskan Skyhigh, Thermo Fisher Scientific, Waltham, MA, USA). For the calibration curve, (+)-catechin was used, with a correlation coefficient (R^2^) of 0.9957. The results were expressed in mg equivalents of (+)-catechin per gram of dry weight.

### 2.10. Flavonoids

The methodology of Ramírez-Jiménez et al. [23] was followed. Fifty μL of extract was combined with 180 μL of methanol and 20 μL of 1% 2-aminoethyldiphenylborate. The absorbance was read at 404 nm using a previously calibrated microplate spectrophotometer (Multiskan Skyhigh, Thermo Fisher Scientific, Waltham, MA, USA). A calibration curve with rutin was used, yielding a correlation coefficient (R^2^) of 0.9971, and the results were expressed in mg rutin equivalents per gram of dry weight.

### 2.11. Antioxidant Capacity

This parameter was determined by two methods: the ferric reducing antioxidant power (FRAP) [24] and the 2,2-azino-bis (3-ethylbenzothiazoline-6-sulfonic acid) method (ABTS) [25].

The FRAP method was performed using the methodology described by Fernandes et al. [24] with slight modifications. To obtain the FRAP reagent, 10 mL of acetate buffer was mixed (30 mM, pH 3.6), 1 mL of 20 mM FeCl_3_ 6H_2_O solution, and 1 mL of 10 mM Ferric 2,4,6-Tripyridyltriazine (TPTZ) reagent. Then 25 μL of extract and 175 μL of FRAP reagent were added to continue with absorbance readings at 595 nm using a previously calibrated microplate spectrophotometer (Multiskan Skyhigh, Thermo Fisher Scientific, Waltham, MA, USA) at different times: 0, 4, 10, 30, 60, and 90 min. The calibration curve was obtained by linear regression from ten levels of Trolox concentration measured in triplicate, with a correlation coefficient (R^2^) of 0.9914. The results were expressed as µmol Trolox equivalents per gram of dry sample.

The methodology described was followed by Álvarez-Chávez et al. [25] methodology with slight modifications. 230 μL of the prepared ABTS solution was added to 20 μL of the extract. The absorbance was read at 520 nm using a previously calibrated microplate spectrophotometer (Multiskan Skyhigh, Thermo Fisher Scientific, Waltham, MA, USA) at different times: 0, 6, 10, 30 and 90 min. The calibration curve was obtained by linear regression from ten levels of Trolox concentration measured in triplicate, with a correlation coefficient (R^2^) of 0.9962. The results were expressed as µmol Trolox equivalents per gram of dry sample.

### 2.12. Phenolic Profile Quantification

Phenolic compound profiling was performed using HPLC-DAD (1200 Series, Agilent Technologies, Palo Alto, CA, USA) as described by Ramírez-Jiménez et al. [26] with modifications. Samples were filtered through 0.20 μm cellulose filters. A ZORBAX Eclipse XDB-C18 column (Santa Clara, CA, USA) (4.6 × 150 mm, 5 µm) was used at a constant flow rate of 0.75 mL/min, with 20 μL injected at a maximum pressure of 600 bar and maintained at a temperature of 30 °C. HPLC-grade water and HPLC-grade acetonitrile, both acidified with 1% formic acid, were used as mobile phases. Solvent A corresponding to acidified water and solvent B to acidified acetonitrile, the following gradient was used: 0.0 min [A: B 95/5], 5.0 min [A: B 85/15], 7.0 min [A: B 75/25], 10 min [A: B 70/30], 12 min [A: B 65/35], 15 min [A: B 60/40], 20 min [A: B 50/50], 25 min [A: B 10/90], 30 min [A: B 5/95], 31 min [A: B 50/50] and 32 min [A: B 95/5]. For the detection of the compounds, a diode array detector (DAD) was used at the following wavelengths: 240, 250, 260, 280, 320, and 330 nm. The autosampler temperature was maintained at 20 °C during the analysis. For identification, the retention times of commercial standards (ellagic acid, gallic acid, catechin, epicatechin, hesperidin, rutin, p-coumaric acid, quercetin, naringenin, and caffeic acid) were compared with those obtained from the samples. For quantification, calibration curves of the commercial standards were prepared in the range of 2.5–12.5 µg/mL, showing R^2^ values between 0.95 and 0.999. The results were processed using ChemStation for LC 3D Systems software (B.04.02 SP1) (Agilent Technologies, Palo Alto, CA, USA).

### 2.13. In Vitro Digestibility of Proteins in Samples

Following the method INFOGEST reported by Brodkorb et al. [27] with slight modifications. Electrolyte solutions for each phase of the test were previously prepared according to the concentrations described in the method: KCl (0.5 mol/dm^3^), KH_2_PO_4_ (0.5 mol/dm^3^), NaHCO_3_ (1 mol/dm^3^), NaCl (2 mol/dm^3^), MgCl_2_(H_2_0)_6_ (0.15 mol/dm^3^), (NH_4_)_2_CO_3_ (0.5 mol/dm^3^), HCl (6 mol/dm^3^) y CaCl(H_2_0)_2_ (0.3 mol/dm^3^). In Eppendorf tubes, 0.5 g of fresh sample was weighed in triplicate, i.e., one tube for each phase of digestion (oral, gastric, and intestinal) to obtain aliquots of each phase for analysis. Duplicates were obtained with two independent experiments. The weighed sample was dissolved in 1 mL of distilled water and stirred to homogenize.

1 mL of the solution called “Oral Mastermix” obtained through the website http://www.proteomics.ch/IVD/ (accessed on 15 June 2025) is programmed according to the number and quantity of samples submitted for analysis. This solution contained α-amylase from human saliva (500 UI/mg, Sigma-Aldrich, St. Louis, MO, USA), the corresponding electrolyte mixture, and a pH of 7. The tubes were then incubated in a dry bath at 37 °C for 2 min. The tubes were then placed in a water bath at 57 °C for 2 min to achieve thermal shock and inactivate the enzymes. The oral phase tubes were then kept frozen at −20 °C for subsequent analysis.

The total volume of the remaining tubes was adjusted to 1 mL, and 1 mL of “Gastric mastermix” solution, obtained from the website http://www.proteomics.ch/IVD/, was added, programmed according to the number and quantity of samples submitted for analysis. The solution contained pepsin from porcine gastric mucosa (400 UI/mg, Sigma-Aldrich, St. Louis, MO, USA), lipase from porcine pancreas (100–500 UI/mg, Sigma-Aldrich, St. Louis, MO, USA), an electrolyte mixture and, pH 3. They were homogenized and incubated in a dry bath at 37 °C for 2 h. After the incubation time, the procedure described in the oral phase for enzyme inactivation and storage of the tubes was repeated.

For the intestinal phase, the procedure described in the previous paragraph was repeated, with the addition 1 mL of the “Intestinal mastermix” solution, obtained from the website http://www.proteomics.ch/IVD/. This solution contained pancreatin from porcine pancreas (8 × USP, Sigma-Aldrich, St. Louis, MO, USA), bile extract from porcine (Sigma-Aldrich, St. Louis, MO, USA), an electrolyte mixture, and a pH of 7. The tubes were homogenized and incubated in a dry bath at 37 °C for 2 h. Finally, the procedure for enzyme inactivation and sample storage was repeated.

After the in vitro digestion procedure was completed, the protein content was determined by the colorimetric method Cabrera-Ramírez et al. [28] with slight modifications. The digested samples were centrifuged, and the supernatant was separated, then adjusted to a pH of around 7 ± 0.5. A stock solution of bovine serum albumin (BSA) 10 mg/mL was prepared, and a calibration curve was created using the following concentrations: 1400, 1200, 900, 600, 300, 150, 60, 30, 10, and 1 µg/mL. After that, a 1:3 sample: Bradford reagent ratio (50 µL of the supernatant/BSA solution + 150 µL Bradford reagent) was placed in a 96-well microplate and left to react for 5 min in the dark. After this time, it was read in a spectrophotometer at 595 nm. The results were expressed as a percentage of digestibility.

### 2.14. Statistical Analysis

All experiments were performed in triplicate, and the results were expressed as mean ± standard deviation. Prior to analysis, data were checked for normality (Shapiro–Wilk test) and homogeneity of variance (Levene’s test) to verify compliance with ANOVA assumptions. One-way analysis of variance (ANOVA) was conducted, and significant differences among means were determined using the Tukey–Kramer multiple comparison test at a significance level of *p* < 0.05. When the assumptions of normality or homogeneity were not met, data were analyzed using the nonparametric Kruskal–Wallis test. All statistical analyses were performed using Minitab 16^®^ (Minitab LLC, Pennsylvania, PA, USA).

## 3. Results

### 3.1. Sensory Analysis

**Figure 1:**
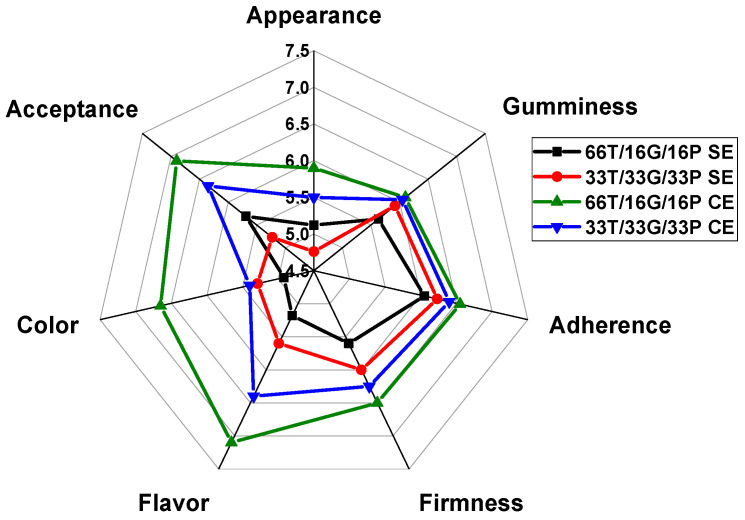
Evaluation of sensory attributes in samples developed with and without sweetener. T = tamarind. G = chickpea flour. P = pineapple, SE = without sweetener, CE = with sweetener.

Among the sensory attributes evaluated by the panelists were appearance, gumminess, adherence, firmness, flavor, color, and overall acceptance. Figure 1 shows the ratings given to each attribute in the samples developed with and without a sweetener.

At first glance, among the samples without added sweetener, the 33T/33G/33P SE formulation received a higher score than the 66T/16G/16P SE sample. However, regarding overall acceptance, the 66T/16G/16P SE formulation achieved a slightly higher mean score compared to the 33T/33G/33P SE formulation. To assess the impact of sweetener addition on sensory perception, the formulations were reformulated with monk fruit sweetener and subjected to a second sensory evaluation. As shown in Figure 1, the sweetened sample 66T/16G/16P CE received the highest acceptance scores among consumers, with mean values close to 7.

To corroborate whether there are significant differences between the scores of each of the samples, the non-parametric Kruskal–Wallis test was applied (Table 2).

Since the *p*-value was >0.05 for the unsweetened samples, there was no significant difference between the scores for each attribute of the two samples. On the other hand, in the sweetened samples, a significant difference (*p* < 0.05) was observed in the color scores. This difference may be due to the higher amount of tamarind added to the 66T/16G/16P formulation. The panelists did not observe significant differences for the other sensory attributes.

For comparison purposes, the same nonparametric test was performed on samples with and without sweetener for each sensory attribute (Table 3).

Significant differences (*p* < 0.05) were found in color and overall acceptance when comparing the sweetened and unsweetened samples. Showed that a higher tamarind content created a more attractive color for the panelists; however, there were no significant differences in flavor (*p* < 0.05).

### 3.2. Bromatological Characterization of Confectionery Products and Commercial Sample

The bromatological characterization of the selected samples is shown in Table 4. The developed confections exhibited significant differences compared to the control sample. There were no significant differences in ash content among the developed confections. About protein content, the sample with 33T/33G/33P obtained the highest percentage at 13.59 ± 0.58%, followed by the sample with 66T/15G/16P at 7.11 ± 0.37% and finally, the control sample at 0.53 ± 0.05%. The percentage of fat was higher (0.48 ± 0.03%) in the sample with 33T/33G/33P than in the sample with 66T/16G/16P (0.19 ± 0.004%).

Regarding carbohydrate content, the 66T/16G/16P sweet had a higher percentage (81.56 ± 0.52%) than the 33T/33G/33P sweet (73.69 ± 1.31%). However, the control sample had a higher carbohydrate content than the developed sweets, at 97.24 ± 0.11%, since its formulation primarily contains sucrose.

The total, soluble, and insoluble fiber content among the developed sweets did not show significant differences, however higher fiber content was evident compared to the control sample. Regarding reducing sugars (Table 5), the 66T/16G/16P candy showed a higher percentage (4.69 ± 0.16%) than the 33T/33G/33P sample (2.93 ± 0.16%) and the control sample (3.42 ± 0.17%). Regarding total soluble solids, no significant differences were observed between the developed candy and the control sample, which obtained the highest value, with 97.50 ± 4.33 °Brix. This result can be mainly attributed to its higher sucrose content. Total soluble solids comprise compounds such as organic acids, pectin, amino acids, and sugars, including glucose, fructose, and sucrose [29].

### 3.3. Phytochemical Characterization and Antioxidant Capacity

**Figure 2:**
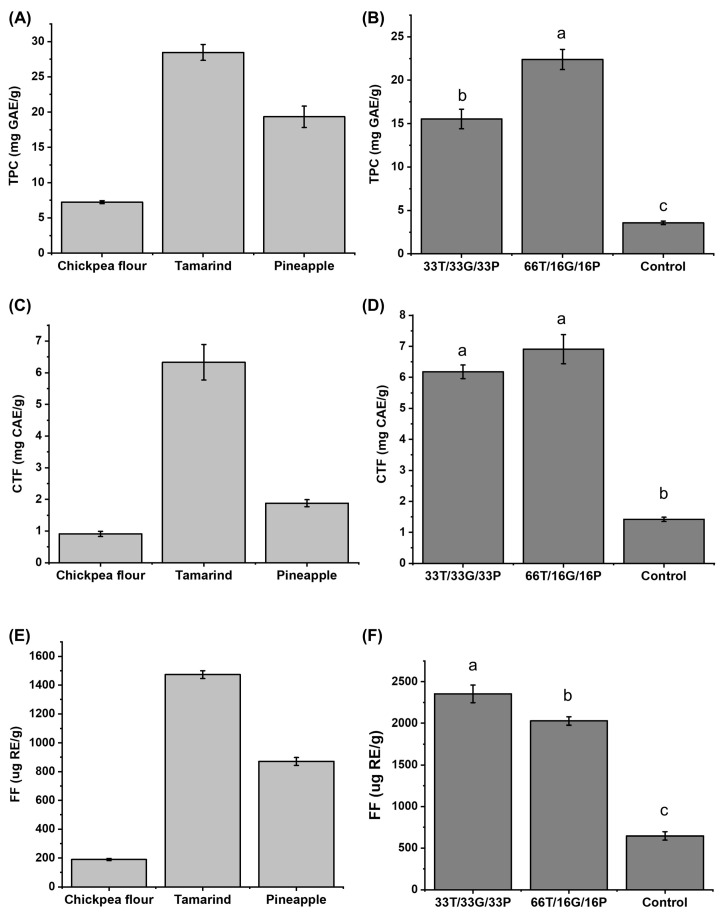
Concentration of total phenolic compounds: (**A**) raw material, (**B**) developed confectionery; condensed tannins: (**C**) raw material, (**D**) developed confectionery and free flavonoids: (**E**) raw material, (**F**) developed confectionery. Different letters in the same column are significantly different in Tukey’s test (*p* < 0.05). GAE = gallic acid equivalents/g of sample on a dry basis. CAE = catechin equivalents/g of sample on a dry basis. RE = rutin equivalents/g of sample on a dry basis. T = tamarind. G = Chickpea flour. P = Pineapple. Control = commercial sample. TPC = Total phenol compounds. CTF = Free condensed tannins. FF = Free flavonoids.

Regarding total phenolic compounds content (Figure 2A), tamarind has been reported to contain content within the range of 4.8 mg GAE/g [30] to 23.70 mg GAE/g [18]. The content of pineapple is reported at 7.29 mg GAE/g dry weight [31] and 37.56 mg GAE/g dry weight [32], so the value obtained in this study falls within the range reported in the literature. The variation in the concentration of the compounds depends on factors such as environmental conditions and the degree of the fruit ripening [30]. On the other hand, the concentration obtained in chickpea (7.24 ± 0.21 mg GAE/g) agrees with the intervals reported by Zhao et al. [33] and Sreerama, Sashikala & Pratape [34], being 1.67 mg GAE/g to 10.84 mg GAE/g.

While for free condensed tannins (Figure 2C), chickpeas have reported contents ranging from 0.23 to 3.17 mg catechin/g [35,36], there is little information for pineapple and tamarind pulp, given that interest is focused on their by-products (seeds and peel). Therefore, the data obtained in this study are the first to be reported to date.

For free flavonoids (Figure 2E), the data obtained are within those reported in the literature, with the following values: chickpeas 100–313.9 µg rutin/g [9,33], tamarind 330–184,000 µg rutin/g [37,38], and pineapple 60–270 µg rutin/g [39].

Tamarind was the ingredient with the highest concentration of phenolic compounds (28.46 ± 1.13 mg GAE/g). Accordingly, in Figure 2B, the formulation with the highest proportion of this fruit (66T/16G/16P) exhibited a higher total phenolic content (22.39 ± 1.16 mg GAE/g) compared with sample 33T/33G/33P (15.53 ± 1.13 mg GAE/g). No significant differences (*p* > 0.05) were found in free condensed tannin content between the two developed formulations according to one-way ANOVA followed by Tukey–Kramer’s test, although both differed significantly (*p* < 0.05) from the commercial control sample.

Theoretically, based on the concentration of phenolic compounds in the raw materials, the confection with 33T/33G/33P would have a total phenolic compound content of 18.32 mg GAE/g. In contrast, the experiment obtained a value of 15.53 mg GAE/g. Therefore, these compounds are preserved in the confection at 84.73%. In the case of the confection with 66T/16G/16P, the theoretical concentration would be 23.37 mg GAE/g, while the experimental concentration obtained was 22.39 mg GAE/g; therefore, a 95.80% conservation.

The 33T/33G/33P sample preserved 91.56% of the condensed tannins, while the 66T/16G/16P sample preserved 95.93%. On the other hand, 93.69% of the free flavonoids remained in the 33T/33G/33P sample and 85.64% in the 66T/16G/16P sample. High-performance liquid chromatography (HPLC) analysis will complement the obtained values.

Furthermore, it was observed that flavonoids (Figure 2F) were lost less in the sample with the highest chickpea content (33T/33G/33P).

**Figure 3:**
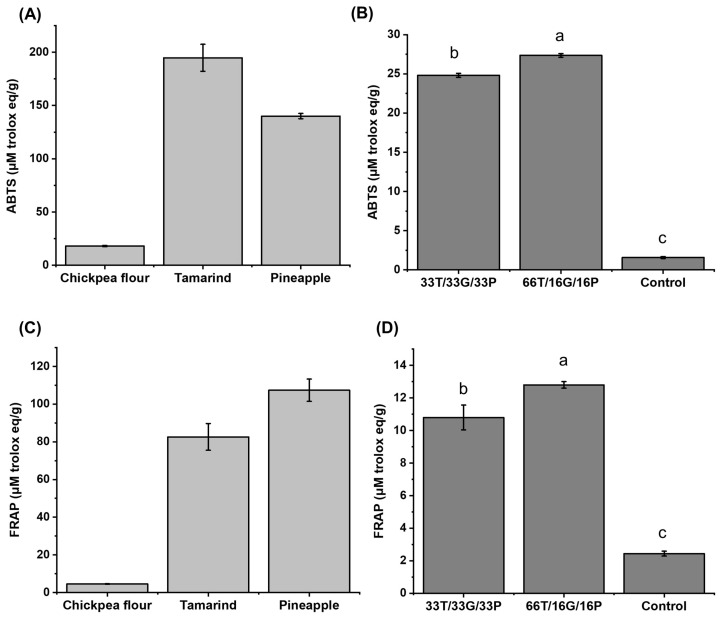
Antioxidant capacity by ABTS: (**A**) raw materials, (**B**) developed confectionery and FRAP: (**C**) raw materials, (**D**) developed confectionery. Different letters in the same column are significantly different in Tukey’s test (*p* < 0.05). T = tamarind. G = Chickpea flour. P = Pineapple. Control = commercial sample.

Subsequently, the antioxidant capacity of the same samples was determined using the ABTS and FRAP methods. The results are shown in Figure 3.

In Figure 3A, for the case of chickpea, Quintero-Soto et al. [40] declare 2.78–27.17 μM Trolox eq/g, being a similar value to that obtained in this work (18.03 ± 0.55 μM Trolox eq/g). Paute & Guamán [41] report values for tamarind between 9.69 and 45.76 μM Trolox eq/g; on the other hand, Paéz-Peñuñuri et al. (2016) [42] report values of up to 1753.8 μM Trolox eq/g. However, in the case of pineapple pulp, information on its antioxidant capacity is scarce, as the study primarily focuses on the byproducts obtained from its processing (mainly peel), with an average concentration of around 88.22 μM Trolox eq/g [43].

Regarding the antioxidant capacity of tamarind using the FRAP method (Figure 3C), Paute & Guamán [41] reported values ranging from 13.10 to 35.43 μM Trolox eq/g, while Dávila-Hernández et al. [18], reported values of up to 326 μM Trolox eq/g. Regarding pineapple pulp, the value obtained (107.35 ± 5.92 μM Trolox eq/g) is within the range reported by Lourenço et al. [44] (51.19–174.50 μM Trolox eq/g). In the case of chickpeas, determined by this same method, the value obtained (4.57 ± 0.15 μM Trolox eq/g) is like the values reported by Kaur et al. [35], where they evaluated flours from different chickpea genotypes, and to those reported by Estanech et al. [45], with ranges of 2.74–29.14 μM Trolox eq/g.

These data will be supplemented after high-performance liquid chromatography (HPLC) analysis, which will be described later.

The 66T/16G/16P confectionery exhibited the highest antioxidant capacity in the ABTS (Figure 3B) and the FRAP (Figure 3D). This could be due to the amount of phenolic compounds, as it was the sample with the highest concentration (22.39 ± 1.16 mg GAE/g) compared to the 33T/33G/33P sample (15.53 ± 1.16 mg GAE/g). Therefore, the results obtained will be complemented with a profile of phenolic compounds determined by HPLC, described in the following section.

The antioxidant capacity of the developed confectionery was lower in both methods than that of the raw materials (Figure 3A,C).

### 3.4. Individual Determination of Phenolic Compounds

Upon the HPLC phenolic profile, Table 6 shows the concentration of different phenolic compounds in the methanolic extracts of the raw materials, developed confectionery and control samples.

In the chickpea flour chromatogram, several peaks were detected; however, based on the available reference standard, only naringenin could be positively identified, confirmed, and quantified by retention time and spectral matching. This finding aligns with the report by Mekky et al. [46], who also reported the presence of naringenin in chickpea samples. In addition to naringenin, traces of p-coumaric acid, hesperidin, syringic acid, and quercetin were detected but not quantified, as their signals were below the quantification limit under the conditions used in this study. De Camargo et al. [47] indicate the presence of p-coumaric acid at concentrations around 16–22 µg/100 g and syringic acid between 0.6 and 1.2 µg/100 g. Meanwhile, Pérez-Ramírez et al. [48] report a quercetin concentration in chickpea sprouts ranging from 0.06 to 0.19 mg/g dry flour. These quantities are not quantifiable under the conditions used in this study.

Regarding tamarind, several peaks were observed in the chromatogram; however, based on the available reference standards, rutin and epicatechin were the only compounds that could be identified, confirmed, and quantified. In addition, traces of p-coumaric acid and syringic acid were detected but not quantified. Essam et al. [49] reported a rutin concentration of 48.74 mg/kg of dry tamarind and described pyrogallol, benzoic acid, and catechin as the predominant phenolic compounds. It is worth mentioning that most studies on tamarind phenolics focus on peel and seeds, where the main compounds reported include procyanidins, epicatechin, and catechin [50,51].

In pineapple pulp, only ellagic acid, catechin, epicatechin, hesperidin, and rutin could be identified, confirmed, and quantified. Likewise, traces of p-coumaric acid, naringenin, and caffeic acid were detected but not quantified. Li et al. [52] reported catechin and epicatechin concentrations of 0.58 mg/g and 0.5 mg/g, respectively, while Sayago-Ayerdi et al. [53] reported values of 0.64 and 0.38 mg/g fresh weight for these compounds. The higher values obtained in this study may be attributed to a concentration effect caused by sample dehydration during preparation or the hydrolysis of polymerized phenolic compounds [54]. Sanahuja et al. [55] also reported a low concentration of coumaric acid (0.001 mg/g), consistent with the levels detected but not quantified in the present study.

In the formulation with the same proportion of ingredients (33T/33G/33P), it was observed that compounds such as catechin (2.72 ± 0.72 mg/g) and epicatechin (5.82 ± 0.52 mg/g) were still present in the mixture. In the formulation with a higher percentage of tamarind (66T/16G/16), epicatechin (53.22 ± 2.68 mg/g) was found in higher concentrations, along with traces of catechin, and rutin were found in higher concentrations. Traces of p-coumaric acid and syringic acid were present in both formulations.

The highest concentration of epicatechin was observed in sample the 66T/16G/16P formulation (53.22 ± 2.68 mg/g) compared to the 33T/33G/33P sample (5.82 ± 0.52 mg/g). This difference is attributed to the high proportion of tamarind, which was the raw material with the greatest concentration of this compound (76.80 ± 7.5 mg/g), followed by pineapple (25.98 ± 0.72), while it was not detected in chickpea flour (Not Detected). Conversely, catechin showed the opposite trend: the 33T/33G/33P formulation exhibited a higher concentration (2.72 ± 0.72 mg/g) than 66T/16G716P sample (5.82 ± 0.52 mg/g), reflecting the contribution of pineapple, the ingredient with the highest catechin content (3.27 ± 0.16 mg/g) whereas tamarind and chickpea flour showed non-detectable levels.

Overall, the formulation with 66T/16G/16P presented 5 phenolic compounds, while 33T/33G/33P contained 4 compounds. These findings are consistent with the total phenolic content (Figure 2) and antioxidant capacity (Figure 3), where 66T/16G/16P exhibited the highest values (22.39 ± 1.16 mg GAE/g), together with greater antioxidant capacity measured by ABTS (27.36 ± 0.23 μM Trolox eq/g) and FRAP (12.80 ± 0.20 μM Trolox eq/g). In contrast, the 33T/33G/33P sample showed a higher concentration of flavonoids (990.88 ± 21.56 μg RE/g) than the 66T/16G/16P sample.

These results demonstrate that the developed confectionery represents a matrix with functional potential, as several phenolic compounds and flavonoids from the raw materials were identified and preserved after processing. However, further in vivo studies are required to confirm these functional properties.

### 3.5. In Vitro Digestibility of Proteins in Samples

**Figure 4:**
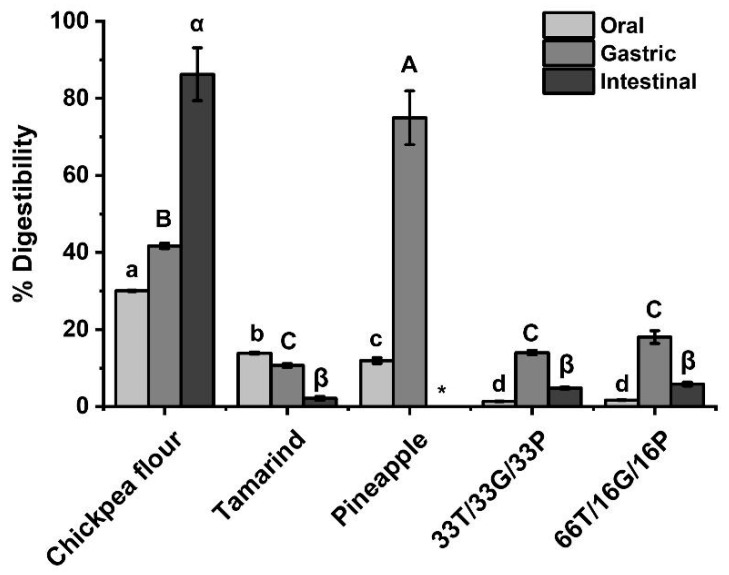
In vitro digestibility of raw materials and developed confectionery. Different letters at the same stage are significantly different in Tukey’s test (*p* < 0.05). T = tamarind. G = Chickpea flour. P = Pineapple. * = below the detection limit.

The digestibility profile of each raw material and the developed confections exhibited distinctive trends throughout the simulated digestion (Figure 4).

For chickpea flour, the digestibility increased progressively from the oral to the intestinal phase. During the oral phase, only α-amylase is present, a non-proteolytic enzyme that acts on internal α-1,4-glycosidic bonds [56]; however, chickpea flour already exhibits approximately 30% digestibility at this stage. This initial increase may be attributed to the release of soluble nitrogenous compounds and partial hydration of proteins; processes that improve protein solubility and enhance enzyme accessibility in the early stages of digestion [57,58,59].

As digestion progressed, the digestibility percentage of chickpea flour increased steadily until reaching 86% in the intestinal phase, consistent with previously reported values for chickpeas (34% to 89%) [9].

In contrast, tamarind pulp exhibited a decreasing trend in digestibility as the digestion phases progressed. Most available studies focus on the analysis of its byproducts, namely the peel and seed [60]; therefore, reports addressing the in vitro protein digestibility of the pulp itself remain scarce.

For the pineapple pulp, digestibility increased markedly from the oral phase to the gastric phase by 75%. However, in the intestinal phase, the protein content was extremely low, below the detection limit. This outcome is consistent with the low initial protein concentration of pineapple pulp (<1–5% dry weight) and its high fiber content, which can limit enzyme access and protein recovery [61,62,63]. In addition, the soluble protein fractions in pineapple pulp are highly susceptible to enzymatic hydrolysis, as previously reported for pineapple byproducts [61], which may further explain the minimal residual protein detected at the intestinal stage. Available studies rarely address the in vitro protein digestibility of pineapple pulp, as most analyses focus on its byproducts, such as peel and crown [64].

In the developed confections, the digestibility percentage decreased considerably in all digestion phases compared to the raw materials. This reduction can be attributed to the complex food matrix and the interactions among ingredients, as digestibility is strongly influenced by the structural and compositional characteristics of the product [65]. From the oral to the gastric phase, a slight increase in digestibility was observed, followed by a marked decline during the intestinal phase. Such behavior may be associated with the formation of protein-phenolic complexes, where polyphenols from tamarind and pineapple interact with legume proteins through hydrogen bonding and hydrophobic interactions, decreasing protein solubility and limiting the access of digestive enzymes. These interactions have been widely reported in food containing plant proteins and phenolic compounds, leading to reduce in vitro protein digestibility [59,66,67].

## 4. Discussion

In the sensory analysis, according to the criteria described by Samakradhamrongthai et al. [68], scores around 7 reflect a high level of acceptability in new product development. In contrast, a score of 6 indicates moderate but not enthusiastic acceptance. In our study, the higher acceptance of sample 66T/16G/16P was statistically significant (*p* < 0.05, one-way ANOVA followed by Tukey–Kramer’s test) and can be attributed to its higher tamarind content, which enhanced the color and flavor of the product. Moreover, its greater sweetness likely contributed positively to overall consumer preference.

Similarly, Kiranmai et al. [69] reported that in gummies made from tamarind with added mango pulp, a higher proportion of tamarind increased product acceptability, emphasizing the influence of sweetness on consumer preference. This suggests that developing healthier confectionery products that reduce refined sugar while maintaining desirable sweetness remains a major innovation challenge.

In the proximate analysis, the increase in protein content between samples 33T/33G/33P and 66T/16G/16P was statistically significant (*p* < 0.05). It was mainly attributed to the percentage of chickpea flour in the formulation, which contained the highest protein content among the ingredients (19.80 ± 0.44%). The higher fat content was also associated with the proportion of chickpea flour, which contained 6.13 ± 0.19% lipids. Conversely, the increase in carbohydrates was attributed to the tamarind content, as this fruit showed higher carbohydrate levels (88.17 ± 0.71%) compared to pineapple and chickpea. Overall, both formulations exhibited significantly higher protein and fiber contents (*p* < 0.05) than the commercial control, supporting their potential as healthier alternatives to conventional confectionery products.

The increase in total phenolic compounds observed in the formulation enriched with tamarind was consistent with the findings of Oluwasina et al. [70], who reported that phenolic content increased proportionally with tamarind addition. The slight decrease in phenolic compounds between the developed formulations was not statistically significant (*p* > 0.05) and may be attributed to the use of mild processing temperatures (<70 °C). Previous studies indicate that phenolic degradation intensifies at temperatures above 70 °C or with prolonged heating [71].

Likewise, the more minor flavonoid loss in sample 33T/33G/33P was likely due to the higher chickpea content, since legume proteins can form non-covalent complexes with phenolic compounds [67], potentially reducing oxidative degradation. Further studies are needed to confirm the occurrence of such interactions during candy processing.

The results of the antioxidant capacity may differ due to various factors, including the raw materials used and development of the confectionery [72], as well as the hydrophilic or lipophilic nature of the phenolic compounds present in the sample [73].

The decrease in antioxidant capacity in the developed confectionery products compared to the raw materials could be attributed to the mixture of the three ingredients and possibly to the formation of complexes between the phenolic compounds and the proteins present in the chickpeas, since there are studies that report the increase or decrease in antioxidant capacity due to the presence of these complexes [74,75]. However, further studies are required to corroborate the presence of complexes in the developed confectionery products.

The 30% digestibility obtained in the chickpea sample is due not so much to enzymatic action, but to the solubility of chickpea proteins. This legume primarily consists of globulins (approximately 60%), glutenins (25%), albumins (between 8 and 12%), and prolamines [76], which are highly soluble in water [77]. In addition to their solubility, factors such as pH and agitation cause the disintegration of the protein network, promoting the release of these proteins [76]. In tamarind pulp, the decrease in digestibility may be due to the presence of antinutritional factors, such as saponins, trypsin inhibitors, and polyphenols, as the latter have been reported to form complexes with digestive enzymes, inactivating them and decreasing protein digestibility [65]. Returning to the results in Figure 2, tamarind was the raw material with the highest concentration of phenolic compounds, tannins, and flavonoids.

Pineapple is a fruit rich in bromelain [8], an enzyme with high proteolytic activity, as reported by Benito-Vázquez et al. [78]. Therefore, due to the presence of this enzyme, pineapple was the ingredient with the highest percentage of digestibility in the gastric phase compared to the other raw materials and confectionery products developed. Furthermore, according to Colleti et al. [79], the enzyme bromelain exhibits a reduction in its activity under changes in pH, temperature, and the presence of other proteases, suggesting that during the intestinal phase of in vitro digestion, it undergoes denaturation or complete proteolysis. Furthermore, simulated digestion studies show that the residual activity of bromelain after the gastric and intestinal phases can drop below 40% [80], supporting the notion that very little intact bromelain protein remains at the end of the process and is therefore not detectable by the Bradford method.

The digestibility of the developed sweets decreased mainly due to the presence of various compounds that play an essential role in in vitro digestibility, such as phenolic compounds from tamarind peel, chickpea protein, and bromelain from pineapple, in addition to the different pH levels ranging from acidic to alkaline in the gastrointestinal environment, which, according to De Morais et al. [74], affects the protonation of amino acid residues and causes conformational changes in proteins. It is also observed that in the developed sweets, the percentage of digestibility increases from the oral to the gastric phase; however, it decreases in the intestinal phase. The increase in the oral and gastric phases, but the decrease in the intestinal phase, may be a consequence of the formation of protein complexes with phenolic compounds through cross-linking, as these complexes have been reported to be less susceptible to enzymatic action, causing a decrease in protein solubility and digestibility [65,76,81]. On the other hand, the formation of these complexes protects phenolic compounds from gastrointestinal degradation, improving their absorption and antioxidant activity [67,82]. Therefore, the developed sweet can serve as a food matrix to ensure that these compounds are released into the gastrointestinal tract and fulfill their various healthier benefits. However, to verify this, an in vivo study would be needed.

## 5. Conclusions

The functional soft confections, formulated with tamarind, pineapple, and chickpea, successfully met the study’s objectives, demonstrating improved nutritional and functional characteristics compared with commercial control. Both optimized formulations demonstrated good sensory acceptance with scores above 7 on a 9-point hedonic scale (*p* < 0.05), confirming their consumer acceptability.

The confection enriched with tamarind (66T/16G/16P) showed the highest total phenolic content (22.39 ± 1.16 mg GAE/g) and antioxidant capacity by ABTS (27.36 ± 0.23 µM Trolox eq/g) and FRAP (12.80 ± 0.20 µM Trolox eq/g), significantly higher (*p* < 0.05) than the 33T/33G/33P sample and the commercial reference. Conversely, the chickpea-rich formulation (33T/33G/33P) preserved a higher proportion of flavonoids (990.88 ± 21.56 µg RE/g). It exhibited greater protein retention (84.73%) after digestion, supporting the role of legume proteins in stabilizing phenolic compounds.

Statistical analyses (one-way ANOVA followed by Tukey–Kramer test, *p* < 0.05) confirmed that the compositional and antioxidant differences between formulations were significant, except for condensed tannins, where no significant differences (*p* > 0.05) were detected.

The observed reduction in protein digestibility during the intestinal phase suggests the formation of protein–phenolic complexes, which may modulate the bioaccessibility of bioactive compounds. These findings indicate that the studied formulations not only fulfill the proposed objective but also offer a feasible strategy for developing nutrient-enriched, plant-based confectionery products.

Overall, the results support the valorization of tamarind, pineapple, and chickpea pulps as functional ingredients, contributing to the design of healthier confectionery alternatives and promoting a sustainable use of underutilized plant-based resources. Future studies should confirm these interactions through structural analyses and in vivo assays to validate the physiological benefits observed in vitro.

## Figures and Tables

**Table 1:** Experimental mixture design for the functional confectionary.

Sample	Tamarind (%)	Chickpea Flour (%)	Pineapple (%)
1	100	0	0
2	0	100	0
3	0	0	100
4	50	50	0
5	50	0	50
6	0	50	50
7	33	33	33
8	66	16	16
9	16	66	16
10	16	16	66

**Table 2:** Nonparametric Kruskal–Wallis test on samples developed without and with sweetener.

Attribute vs. Sample	Sample	Value *p*	Sample	Value *p*
	Without Sweetener		With Sweetener	
Appearance	33T/33G/33P	0.289	33T/33G/33P	0.371
	66T/16G/16P		66T/16G/16P	
Gumminess	33T/33G/33P	0.253	33T/33G/33P	0.945
	66T/16G/16P		66T/16G/16P	
Adherence	33T/33G/33P	0.184	33T/33G/33P	0.788
	66T/16G/16P		66T/16G/16P	
Firmness	33T/33G/33P	0.096	33T/33G/33P	0.619
	66T/16G/16P		66T/16G/16P	
Flavor	33T/33G/33P	0.147	33T/33G/33P	0.096
	66T/16G/16P		66T/16G/16P	
Color	33T/33G/33P	0.347	33T/33G/33P	0.006
	66T/16G/16P		66T/16G/16P	
Acceptance	33T/33G/33P	0.204	33T/33G/33P	0.109
	66T/16G/16P		66T/16G/16P	

**Table 3:** Nonparametric Kruskal–Wallis test on samples developed without and with sweetener.

Attribute vs. Sample	Sample	Value *p*
Appearance	33T/33G/33P SE	0.141
	66T/16G/16P SE	
	33T/33G/33P CE	
	66T/16G/16P CE	
Gumminess	33T/33G/33P SE	0.570
	66T/16G/16P SE	
	33T/33G/33P CE	
	66T/16G/16P CE	
Adherence	33T/33G/33P SE	0.279
	66T/16G/16P SE	
	33T/33G/33P CE	
	66T/16G/16P CE	
Firmness	33T/33G/33P SE	0.105
	66T/16G/16P SE	
	33T/33G/33P CE	
	66T/16G/16P CE	
Flavor	33T/33G/33P SE	0.0
	66T/16G/16P SE	
	33T/33G/33P CE	
	66T/16G/16P CE	
Color	33T/33G/33P SE	0.005
	66T/16G/16P SE	
	33T/33G/33P CE	
	66T/16G/16P CE	
Acceptance	33T/33G/33P SE	0.001
	66T/16G/16P SE	
	33T/33G/33P CE	
	66T/16G/16P CE	

T = tamarind, G = chickpea, P = pineapple, SE = without sweetener, CE = with sweetener.

**Table 4:** Bromatological characterization of the developed formulations.

Sample	Ash *	Protein *	Lipids *	Carbohydrates *	Total Fiber *	Soluble Fiber *	Insoluble Fiber *
	%						
33T/33G/33P	11.78 ± 0.80 ^a^	13.59 ± 0.58 ^a^	0.48 ± 0.04 ^a^	73.97 ± 1.31 ^c^	32.62 ± 2.38 ^a^	21.01 ± 3.08 ^a^	11.62 ± 0.69 ^a^
66T/16G/16P	11.10 ± 0.08 ^a^	7.11 ± 0.37 ^b^	0.19 ± 0.004 ^b^	81.56 ± 0.52 ^b^	32.55 ± 2.38 ^a^	18.48 ± 0.48 ^a^	11.59 ± 0.69 ^a^
Control	2.18 ± 0.04 ^b^	0.53 ± 0.05 ^c^	0.04 ± 0.004 ^c^	97.24 ± 0.11 ^a^	4.79 ± 0.54 ^b^	3.63± 0.75 ^b^	1.16 ± 0.21 ^b^

Values represent the mean ± standard deviation of three replicates, * dry basis. Different letters within the same column are significantly different in Tukey’s test (*p* < 0.05). T = tamarind. G = Chickpea flour. P = Pineapple. Control = commercial sample.

**Table 5:** Amount of reducing sugars and total soluble solids.

Sample	Reducing Sugars (%)	Total soluble Solids (° Brix)
33T/33G/33P	2.93 ± 0.16 ^c^	80.20 ± 0.15 ^b^
66T/16G/16P	4.69 ± 0.16 ^a^	78.04 ± 0.99 ^b^
Control	3.42 ± 0.17 ^b^	97.50 ± 4.33 ^a^

Values represent the mean ± standard deviation of three replicates. Different letters within the same column are significantly different in Tukey’s test (*p* < 0.05). T = tamarind. G = Chickpea flour. P = Pineapple. Control = commercial sample.

**Table 6:** Concentration of different phenolic compounds in methanolic extracts.

Phenolic Compound [mg/g]	Chickpea Flour	Tamarind	Pineapple	33T/33G/33P	66T/16G/16P	Control
Ellagic acid	ND	ND	2.30 ± 0.21	ND	ND	ND
Catechin	ND	ND	3.27± 0.16 ^a^	2.724 ± 0.72 ^a^	NQ	ND
Epicatechin	ND	76.80 ± 7.5 ^a^	25.98 ± 0.72 ^c^	5.82 ± 0.52 ^d^	53.22± 2.68 ^b^	ND
Hesperidin	NQ	ND	25.45 ± 2.20	ND	ND	ND
Rutin	ND	1.07 ± 0.44 ^b^	2.43 ± 0.04 ^a^	ND	NQ	ND
P-coumaric acid	NQ	NQ	NQ	NQ	NQ	ND
Naringenin	0.89 ± 0.09	ND	NQ	ND	ND	ND
Caffeic acid	ND	ND	NQ	ND	ND	ND
Syringic acid	NQ	NQ	ND	NQ	NQ	ND
Quercetin	NQ	ND	ND	ND	ND	ND

Values represent the mean ± standard deviation of two replicates. Expressed as mg of compound per gram of dry sample. Different letters in the same row correspond to significant differences in Tukey’s test (*p* < 0.05). T = tamarind. G = Chickpea flour. P = Pineapple. Control = commercial sample. ND = Not detected. NQ = Not quantifiable.

## Data Availability

The original contributions presented in this study are included in the article. Further inquiries can be directed to the corresponding author.

## Abbreviations

The following abbreviations are used in this manuscript:

HPLC	High-Performance Liquid Chromatography
ABTS	2,2-azino-bis (3-ethylbenzothiazoline-6-sulfonic acid
FRAP	Ferric reducing antioxidant power
SE	Without sweetener
CE	With sweetener
